# Developing user-friendly ambulatory referrals: a quality improvement study in GI referral services at a large academic safety net hospital system

**DOI:** 10.1186/s12913-025-12976-3

**Published:** 2025-07-01

**Authors:** Aaron Gerard Issac, Wendy Du, Alix Youngblood, Wilhelmina Prinssen, Kelly Carroll, Robert Geller, Palak Patel, Bhavin Adhyaru, Jason Brown

**Affiliations:** 1 Department of Medicine, Emory University School of Medicine, 2015 Uppergate Dr, Atlanta, GA 30307 USA; 2 Grady Health System, Atlanta, GA USA; 3 Department of Pediatrics, Emory University School of Medicine and Grady Health System, Atlanta, GA USA; 4 Department of General Medicine, Emory University School of Medicine and Grady Health System, Atlanta, GA USA; 5 Division of Digestive Diseases, Emory University School of Medicine and Grady Health System, Atlanta, GA USA

**Keywords:** Electronic referrals, Direct access endoscopy, Programmatic colon cancer screening, Electronic medical record, Safety net hospital

## Abstract

**Background:**

In the United States, more than a third of patients are referred to specialists each year; however, most of these referrals do not lead to completed appointments. At the Grady Health System (GHS), our large safety net hospital system, the initial gastroenterology (GI) referral process suffered from multiple inefficiencies, creating barriers to care. We aimed to improve GI referrals with both a triage and a direct-to-endoscopy program to relieve systemic barriers to GI care at GHS especially around colorectal cancer screening.

**Methods:**

Given wait times for GI services and employee dissatisfaction with navigating patients through the referral process, a GI smart order set was built using the Epic electronic medical record. The process took 8 months and included automated anesthesia screening as well as periprocedural guidance on blood thinners. We measured time from placement of referral for screening colonoscopy to scheduling of the screening colonoscopy to assess improvement in wait times for GI services.

**Key results:**

In our pre-implementation survey, 60% of providers placed at least one urgent referral a month, and 55% of providers were either somewhat or very dissatisfied with the referral process. This led to the creation of multiple unofficial and only partially successful bypasses to expedite GI care. With the new GI smart order set, there was a 93% reduction over 12 months in the time from providers screening colonoscopy referral request to procedure scheduling from an average of 422 to 28 days. In addition, overall rates of colorectal cancer screening increased approximately 6% from 43.5 to 49% since the order set was implemented.

**Conclusions:**

This novel outpatient GI referral smart order set addressed multiple barriers to care and created a novel triage mechanism as well as a direct-to-endoscopy referral stream. This model can be used to improve triaging and increase access to GI and other specialist services.

**Supplementary Information:**

The online version contains supplementary material available at 10.1186/s12913-025-12976-3.

## Background

In the United States, more than a third of patients are referred to specialists each year. The specialty referral process has long been a frustrating challenge for both internists and specialists alike as only 35–50% of scheduling attempts result in completed appointments [1]. These low completion rates are often due to systemic infrastructure breakdowns such as low scheduling rates, prolonged wait times, and minimal closed loop communication between referring providers and the specialists [2]. At large safety net hospital systems these already difficult challenges can be amplified as patients often have low levels of healthcare literacy, decreased transportation options, and language barriers. The gastrointestinal (GI) field is no exception to referral challenges, especially considering updated United States Preventive Services Task Force (USPSTF) guidelines recommending earlier onset colon cancer screening at the age of 45. This may lead to increased referrals and place a significant burden on already strained large safety net hospital systems [3].

Electronic referral systems have been used by primary care providers (PCPs) in different health care systems to improve access to specialty care. Kim et al. demonstrated that PCPs at San Francisco General Hospital reported that electronic referrals improved clinical care. Though some providers did find that referrals that took longer than 6 min were less likely to report that this improved clinical care [4]. In our literature review we were unable to find electronic referral studies that studied direct to endoscopy care.

Colorectal cancer (CRC) in the United States is the fourth most common type of cancer and has the second highest mortality rate behind lung cancer. The US population has a 4.1% chance of being diagnosed with colorectal cancer within their lifetime; however, only 37% of patients are diagnosed at a stage where local intervention is possible [5]. Therefore, delayed GI referrals can be devastating for patients who may benefit from early intervention. Providers often believe delays are multifactorial due to misdiagnoses, lack of using referral guidelines, and poor continuity of care; patients believe delays are due to decreased education, lack of social support, and living further away [6]. Electronic-referral solutions have been shown to be helpful in improving access to specialty care, reducing wait times, and improving the quality of referral communications across multiple systems in the North America and Europe [7].

Grady Health System (GHS) is a 900 + bed Level 1 trauma center with 6 neighborhood facilities, a comprehensive Infectious Disease Center, and a Behavioral Health Center. GHS handles over 700,000 patient visits annually and patients come from all parts of Georgia, United States. The flagship hospital for GHS is the fifth largest public hospital in the United States and is in the heart of Atlanta, Georgia [8]. Patients who obtain their care at GHS are often part of underserved communities impacted by multiple social determinants of health, including but not limited to: increased rates of poverty, lower access to healthcare insurance, lower education attainment, language barriers, food insecurity, housing insecurity, and inconsistent transportation [9].

Outpatient Gastroenterology Service operations at GHS previously spanned two separate administrative departments within GHS: Perioperative Services and Ambulatory Care Services. These departments utilize separate staff and separate scheduling programs within GHS’s electronic medical record system that do not communicate and do not shift patients between them. Clinic and procedure schedulers receive requests for appointments via a multitude of referral orders that are redundant and have unintuitive nomenclature. Referrals are sent to scheduling depots that are inconsistently monitored by scheduling staff of both administrative units who often do not have access to the correct scheduling programs to act on these referrals.

Within Perioperative Services, procedure referrals come from Grady primary care clinics, neighborhood primary care clinics, inpatient hospital medicine teams, surgical services, oncology clinics, as well as the emergency department. Patients are often instructed to bring themselves to the GI endoscopy lab to schedule an appointment. Aside from unannounced walk-ins, schedulers also receive Epic chat messages through the Epic system Electronic Medical Record (EMR) as well as Epic inbox messages. Importantly, there is no triage function, meaning routine diagnostic referrals are placed into the same depot as patients with alarm symptoms as an indication for endoscopy. Furthermore, there is no automatic mechanism to determine which patients require preoperative assessment and clearance by anesthesiology, leading to potentially unsafe situations discovered only on the day of procedure. Therefore, patients who have prepared for their procedure are often turned away due to lack of pre-operative clearance. Finally, there is no guidance for referring providers regarding management of anticoagulation and antiplatelet agents, leading to pre-operative confusion and potentially additional cancellations.

Within Ambulatory Care Services, GI operates a trainee clinic, multiple attending clinics, and daily advanced practice provider (APP) clinics. Approximate patient volume is 190 patients per week, including both new referrals and follow-ups. There was a backlog of 2,662 new patient referrals. The wait time for new patient visits was 74 days, and for existing patients, the wait time was 44 days. There was a separate referral depot with an additional 450 patients referred to GI clinic specifically to discuss colorectal cancer screening modalities. As with procedure referrals, there is no triage system for expediting clinic referrals with alarm features. As a work-around, inpatient GI consult teams and individual attendings are routinely contacted 24 h a day to facilitate urgent follow up, despite having no access to place patients on an already overbooked schedule.

## Objective

The creation of an urgent referral process and direct-to-endoscopy referral process would be an innovative first step in relieving severe systemic barriers to care at GHS. The new Epic EMR Smart Order Set would receive, triage, and direct thousands of yearly outpatient GI referrals. Through the novel smart order set, operational baseline data can be gathered to assess referral volume and scheduling efficiency within GI at GHS. This initiative is designed to improve transitions of care among Grady departments to GI, expedite urgent GI referrals, and address severe and urgent health disparities and is a direct attempt to ease systemic barriers. Specifically, we aim to evaluate the colon cancer screening wait times and colon cancer screening rate of patients at GHS using the data reports from our electronic medical record system. so that we can expedite this important preventative care for the entire GHS. If successful, we hope this order set can be used as a model for other large safety net hospitals to improve their own GI and outpatient services and reduce the barriers of the referral process. In our literature search, no other project has attempted to streamline the referral system in this fashion and this is the first to attempt such a large array of encounters based on the indication (i.e. positive fecal immunochemical test (FIT), dysphagia, cirrhosis screening, Barrett’s esophagus surveillance, etc.).

## Methods

This quality improvement study was conducted at GHS, a single tertiary-care center in a large urban setting, from August, 2022 to December, 2023. All patients who had ambulatory procedures or new clinic referrals were part of this study.

### Pre-Implementation survey

**Fig. 1:**
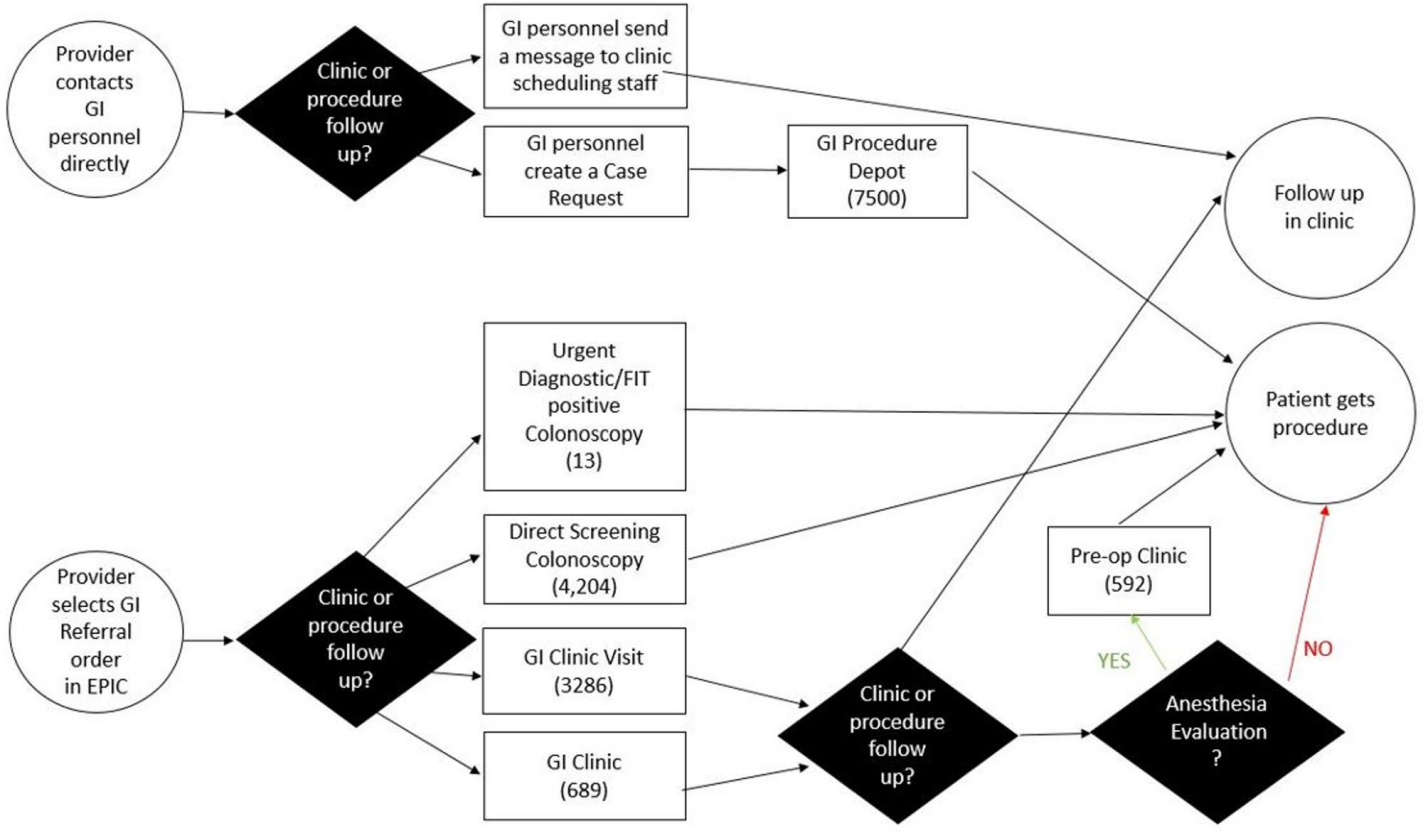
Gastroenterology (GI) Referral Process Map Prior to Implementation of the Order Set. FIT (fecal immunochemical test)

An initial pre-implementation survey was sent out to GHS providers who interacted with the original GI referral process (Fig. 1). This initial process was not streamlined and had both the epic referral and GI providers directly scheduling for clinic and procedure visits without a triaging mechanism. A novel survey was developed for this study (supplement I) and distributed to referring providers at GHS to understand barriers and perceptions of the original GI referral process. The survey consisted of demographic data from the employees to understand their role within GHS, professional degree, and primary specialty. The survey gauged their interaction with GI referral services though the number of referrals placed, and the nature of these referrals including urgency and indication. In addition, the survey gauged employee satisfaction with the original GI referral process with a Likert scale as well as what work-arounds were currently being used to bypass the original GI referral process. The work-around options were a multiple select survey with the most common work arounds as well as an option for free response. The survey results were then used in a framework of “The Model for Improvement” to understand what part of the process needed to be revised.

### Experimental design

A Plan-Do-Study-Act (PDSA) method was implemented to improve access to GI services at GHS. Findings from the pre-implementation survey were used to address provider barriers to patient access to GI services. A new process flow map was created and rolled out with input from a multidisciplinary team and implemented using a new GI referral smart order set. Baseline data on GI referral volumes and scheduling efficiency through this order set was collected.

### Smart order set build

This GI smart order build process took approximately 8 months using Epic Systems as the EMR. Epic is the preferred EMR for more than 2,100 hospitals and 60,000 clinics, with a 2015 report showing 54% of patients in the USA had their medical records on this system [10, 11]. Epic has both inpatient and outpatient interfaces that facilitate referrals between the different departments (i.e., an inpatient referral for an outpatient colonoscopy). The multiple features of Epic also allow a provider to create order sets within a category, which speeds along repetitive orders such as routine health maintenance and cancer screening exams. There are also options to flag certain orders, which can encourage providers to choose alternative options when there are medication shortages or procedural bottlenecks. The work for the smart order set was completed by a GHS Epic build team comprised of Informatics experts as well as representatives from both specialties within GHS IT concentrating on both outpatient clinic referrals and outpatient procedural referrals.

After the go-live for the new smart set, a data dashboard was built within Epic to automatically run monthly administrative reports, collecting data about smart order set usage and referral volumes. The information technology team used Microsoft Power Business Intelligence software which included data from the electronic health record for further analysis. Data that was brought into the software was the following: (1) urgent procedure referral date, scheduled procedure date, and the interval in between, (2) non urgent diagnostic procedure referral date, scheduled procedure date, and the interval in between, (3) urgent clinic referral date, scheduled clinic date, and the interval in between, (4) non urgent clinic referral date, scheduled clinic date, and the interval in between, (5) screening colonoscopy referral date, scheduled procedure date, and the interval in between, (6) FIT positive order date, result date. The primary method to assess whether this intervention was successful was the time from PCP refer for a screening colonoscopy to when it was scheduled. Procedural referrals are categorized by diagnostic or screening indications and clinic referrals and diagnostic indications were further separated into urgent (< 30 days) versus non-urgent as outlined in Table 1.

**Table 1:** Epic dashboard breakdown based on referral types and urgency of indication

**Epic Dashboard Data Breakdown**				
**Procedural referrals**			**Clinic referrals**	
**Diagnostic Indications**		**Screening**	**Urgent**	**Non-Urgent**
**Urgent**	**Non-Urgent**			
Number of urgent procedure referrals per month	Number of nonurgent procedure referrals per month	N/A	Number of urgent clinic referrals per month	Number of nonurgent clinic referrals per month
Number of urgent procedure referrals successfully scheduled for procedure	Number of nonurgent procedure referrals successfully scheduled for procedure		Number of urgent clinic referrals scheduled for clinic visit	Number of nonurgent clinic referrals scheduled for a clinic visit
Average time from urgent procedure referral to procedure scheduling	Average time from nonurgent procedure referral to procedure scheduling		Average time from urgent referral to scheduled clinic visit	Average time from nonurgent clinic referral to scheduled clinic visit

## Results

An initial survey to providers at GHS was sent out prior to the implementation of the new GI smart order set. Baseline data of the 56 participants in the survey showing their role within Grady was collected. Responders included attending faculty physicians, trainees such as residents and fellows, and APPs. Their roles, professional degrees and medical specialties are shown in Table 2.

**Table 2:** Role, professional degree, and primary specialty of survey responders

Position	Numbers
Role within Grady	
Trainees	7
Faculty	41
GHS Employees	8
Professional Degree	
MD	45
MD, in fellowship	2
MD, in residency	6
PA	2
NP	1
Primary Specialty	
Hospitalist	6
PCP	27
Ob/Gyn	4
Medical Subspecialty	14
Neurology	3
Cardiology	1
Surgery	1

**Fig. 2:**
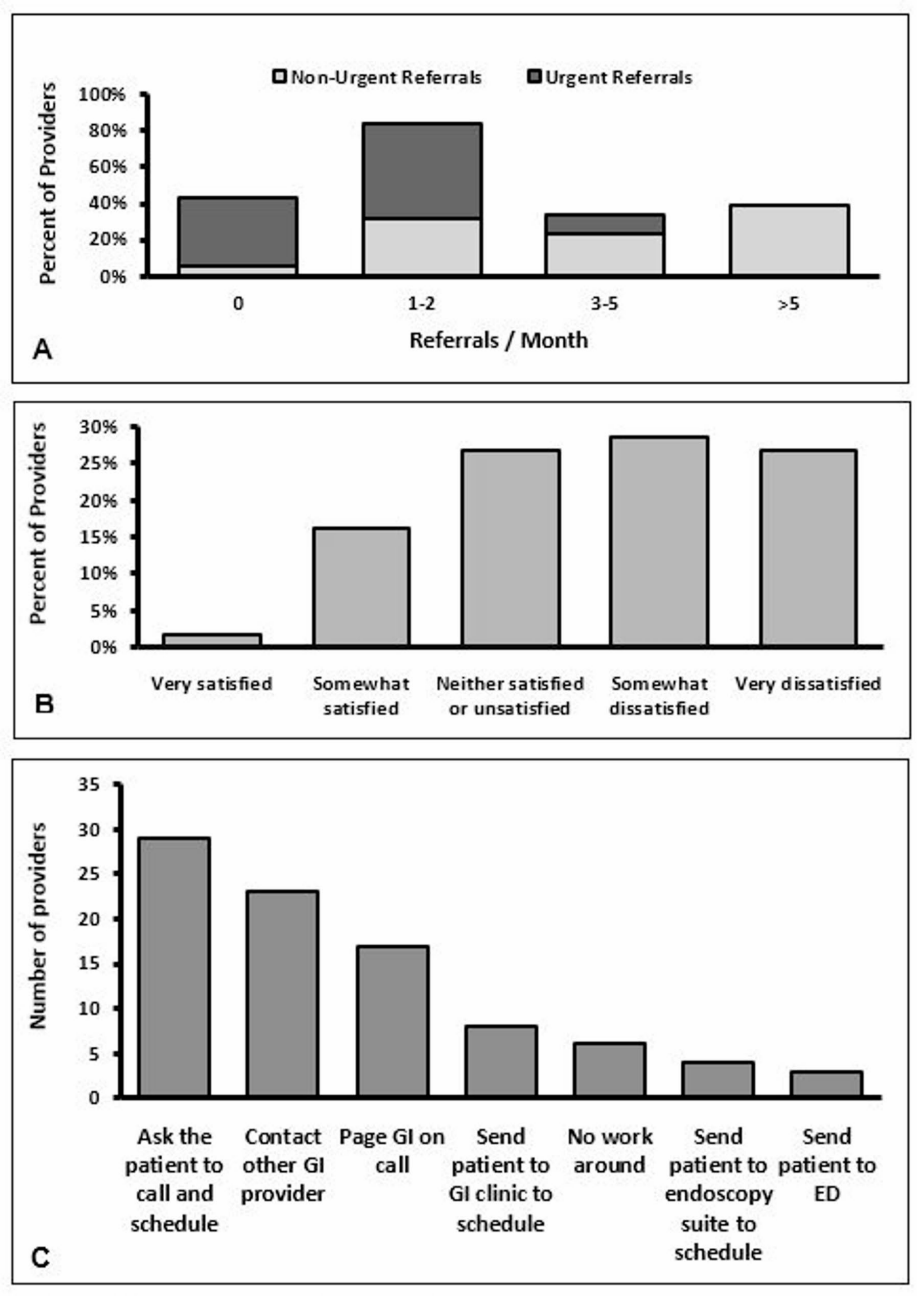
**A** Non-urgent referrals and urgent referrals per provider per month; **B** Provider satisfaction with current referral system; **C** Provider approach to overcoming barriers in the colorectal cancer screening referral process

**Fig. 3:**
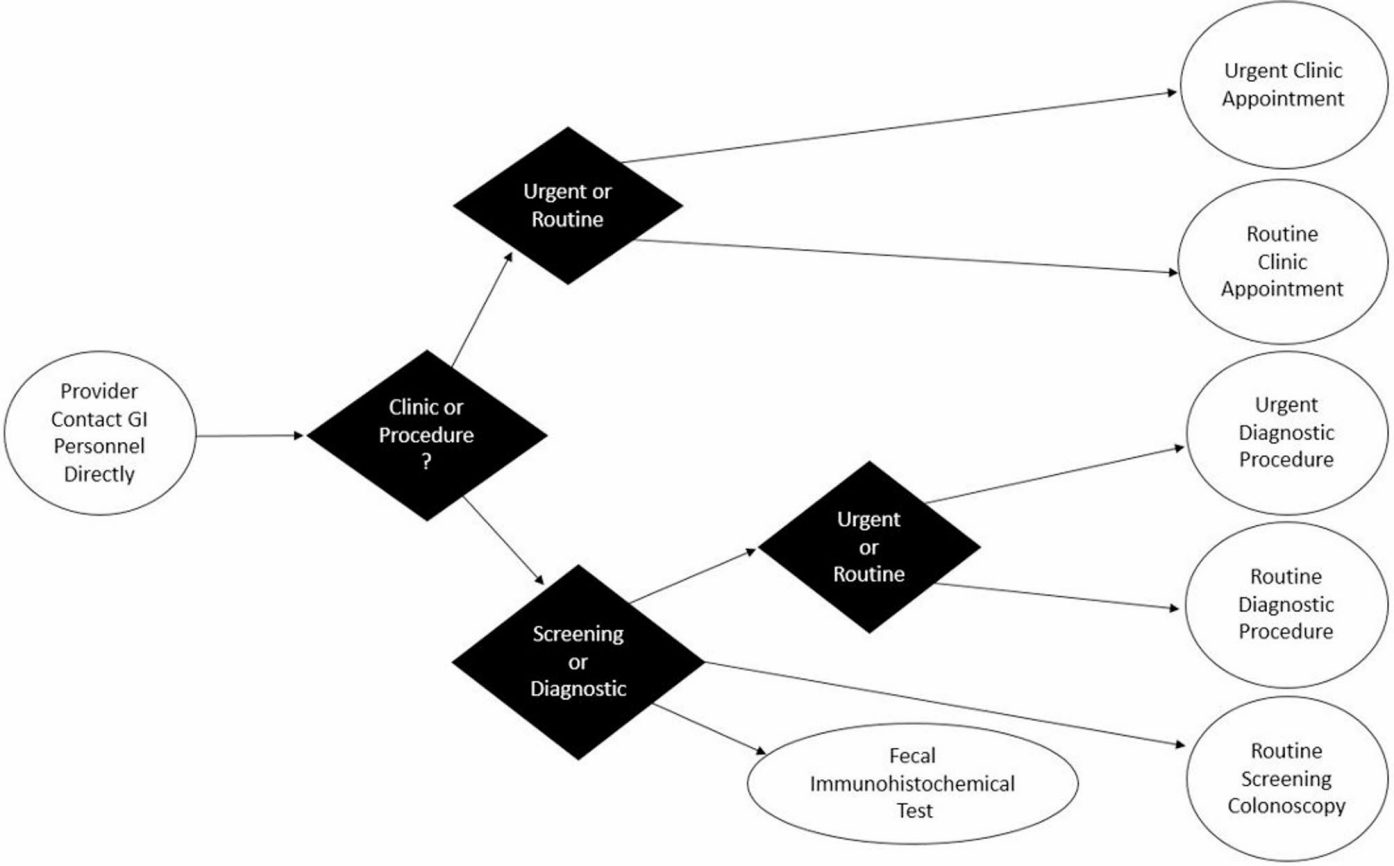
Simplified Gastroenterology (GI) referral process map post implementation of the order set

They were then asked about their interaction with the current system such as the number of referrals that they placed, how many urgent referrals they placed, the reason for referrals, their overall satisfaction with the current system, and any work-arounds they used within their practice (Fig. 2). This showed that 39% of providers placed more than 5 non-urgent referrals, and 52% or providers placed 1–2 urgent referrals per month. In addition, 29% of providers were somewhat dissatisfied and 27% of providers were very dissatisfied with the current process. This led to 29% of providers having patients directly call to schedule, and 23% of providers directly reaching out to GI personal for scheduling assistance. The final order logic is seen in Fig. 3, with procedure indications separated by diagnostic and screening purposes, and then both diagnostic and clinical indications further separated into urgent or routine indications.

The GI referral pathway’s first branch point is whether the provider needs clinic or procedural services. After this, the clinic pathway documents the urgency of the referral and characterizes the type of referral. For the procedural pathway, a second branch point is whether CRC screening is needed or other diagnostic testing. For the CRC screening, the two tier 1 USPSTF tests are embedded with notes about using FIT for average risk patients given endoscopy bottlenecks at GHS. For other diagnostic procedures, the urgency of the referral and common indications can then be selected. Other useful logic in the order set are reminders if a previous referral has been placed for a similar indication. Patients at GHS also often have multiple comorbidities, which leads to higher pre-op American Society of Anesthesiologists (ASA) scores during routine procedures when compared to the general population. For this order set, a careful anesthesia evaluation was incorporated, and anesthesiologists were consulted to build a comprehensive list of comorbidities and conditions. In the order set, these features in the patient’s problem list or active diagnoses would trigger an automatic referral to the Preoperative Anesthesia Testing (PAT) clinic. High-risk patients, as defined by the anesthesia team, will now automatically be scheduled for PAT clinic, and will be required to complete this visit before scheduling their procedure.

**Fig. 4:**
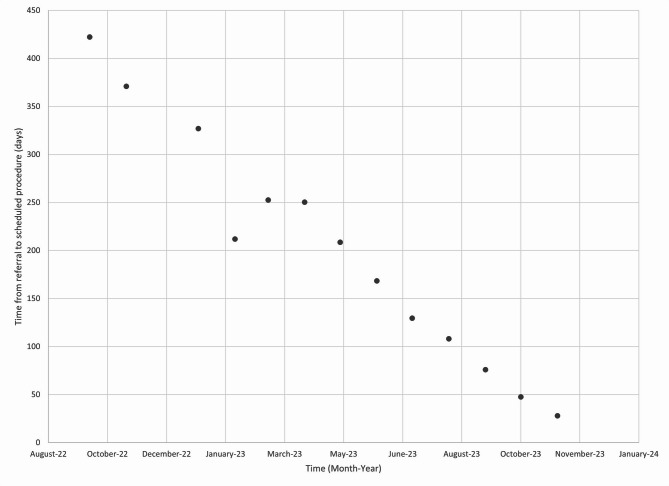
Screening Colonoscopy Referrals: Average Order to Scheduling time

**Fig. 5:**
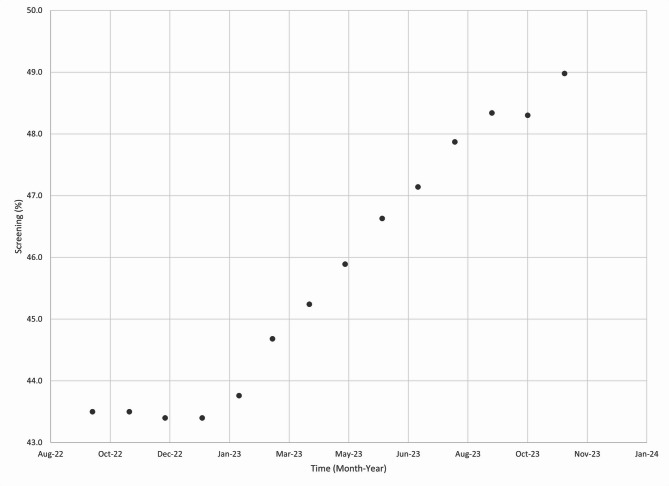
Colorectal Cancer screening rate of patients at Grady Health System

This new order set is designed to improve patient care through streamlining multiple prior workflows and triaging patients for intervention. Initial data from the implementation shows there are clear reductions in average interval between ordering and scheduling new referrals for colon cancer screening colonoscopies (Fig. 4). In October of 2022 the wait time was 422 days for the procedure to be scheduled, with the order set this was brought down to 28 days by November, 2023. Given the procedural backlog for both diagnostic and screening colonoscopies, other modalities such as FIT testing were recommended for average risk patients. The changes were incorporated into the smart order set and led to an overall increase in CRC screening rates at GHS (Fig. 5). The initial screening rate for CRC cancer was 43.5% in October, 2022 and this increased to 49% by November, 2023.

## Discussion

This was the first project to perform a comprehensive redesign of the GI referral workflow at GHS, and to the best of our knowledge did not find similar projects in our literature review. The existing workflow had multiple challenges for patient care and staff support. This led to multiple providers being dissatisfied with the system and work-arounds that relied on providers having more knowledge and connections within the system; even then, care was fragmented and inefficient. Patient care is already limited in large safety net hospitals and the complex GI referral workflow led to difficulty triaging patients as well as large wait times for routine procedures. Both patient and staff challenges led to the conception of this new novel ambulatory referral GI workflow.

There were several logistical challenges that complicated this endeavor. This quality improvement process began at the height of the COVID-19 pandemic, when administrative and healthcare provider focus was on other more urgent issues. Garnering approval and resources for this project required approaching multiple leadership teams from several separate GHS administrative departments. Once approval was granted, a large, coordinated effort was needed to organize regular meetings with quorum to ensure each team was on the same page and had enough participation to afford decision-making authority that was both binding and durable. Also, multiple administrators, healthcare providers, and staff collaborated with two different software Epic build teams to execute this project. The primary driver for this project was a GI physician who worked with multiple leadership teams to address their concerns and incorporate their advice. This project partnered with leadership from Perioperative Services, Ambulatory Care Services, Grady Quality Improvement, Grady Informatics, Grady Information Technology/Epic Support, and the GI Endoscopy Lab to create a new Epic smart order set for all outpatient GI referrals. In addition, outpatient GI had 2 separate departments of perioperative services and ambulatory care services that did not have integrated scheduling systems.

Other challenges included guidance regarding perioperative anticoagulation and antiplatelet therapy. Cardiology, Neurology, and Vascular Surgery assisted leaders from the Anticoagulation Clinic by providing guidance on whether anticoagulation should be held, and if so for how long. This input was incorporated into best practices advisory (BPA) box that automatically fired if anticoagulation or antiplatelet agents were listed on the patient’s active medication list. This BPA assists referring providers in making informed choices regarding periprocedural management and patient instruction for these medications. In cases where guidance is limited, an automatic GI clinic referral is placed instead of a procedural referral, so that GI may review the indication, urgency, and appropriateness of the referral and further coordinate care as necessary.

Prior analysis of delays in GI referrals centers around CRC screening. The main reasons for slow referrals are primarily delayed appointments, though also consult denials, as well as patient access to care and missing appointments. Consult denials were often seen in the setting of ambiguous guidelines that referring providers did not understand [12]. This is directly addressed in our order set by setting out clear indications for interventions. Additionally, backlogs in endoscopy can more meaningfully addressed by offering FIT as a clear alternative, thereby taking steps toward shifting from a purely colonoscopy-based opportunistic screening to a FIT and colonoscopy-based programmatic screening. Other studies at large safety net hospital systems have seen delays in GI care, specifically diagnostic colonoscopies because of decreased care coordination and closed loop communication [13]. We hope to address this with the order set dashboard to keep track of the efficiency of referrals to make real time changes.

Other quality improvement projects that improved cancer screening rates targeted patients, providers, or general community awareness. An urban Federally Qualified Health Center used a fotonovela narrative comic approach to increase awareness to patients, and used text message reminders to increase adherence to FIT testing [14]. Text message reminders was also used by Solonowicz et al. though for colonoscopy prep instructions [15]. Both of these interventions used different technological approaches to increase engagement and screening rates for colon cancer screening in their communities. This approach was also seen in a large multicenter study that worked to increase cancer screening rates to pre COVID pandemic to during the pandemic [16]. Baus et al. used an EMR based approach to address colon cancer screening rates by having clinics work with their EMR vendor and information technology team to increase screening rates [17]. Though the EMR was used to assess for pre and post intervention data, an EMR specific intervention was not utilized.

The main goal of this project is to improve the triaging of patients in need of timely intervention. As the referral system is implemented, there will be a learning and logistical curve. This project allows for real time feedback of the smart order set’s roll out process, and this feedback is readily accessible to leadership. The Perioperative and Ambulatory Care teams can then use the actionable, operational data to consider staffing and funding levels, particularly with attention to the most urgent patients and highest revenue streams (endoscopy procedures). This will be most important in the initial stages when we expect to have a higher volume of diagnostic colonoscopies given increased CRC screening via FIT testing, which could overwhelm GHS endoscopy capacity.

Aside from dynamic changes in staffing by leadership, front-line users can receive feedback at the departmental and even individual level. Providers that are underutilizing FIT exams can be targeted for further education. Providers whose patients have high incompletion rates for either FIT or colonoscopy can also be targeted for further quality improvement efforts, particularly in cases where Anesthesia clearance is not granted, or anticoagulation or antiplatelet agents are not managed appropriately.

Future improvements in the referral order set will be aimed towards closed loop communication and having dedicated scheduling coordinators. Closed loop communication will allow referring providers to be notified if their referral was denied or delayed with an explanation. This will allow providers to either re-refer the patient with an updated referral or get in touch with the GI scheduler to better triage the referral. Dedicated coordinators will be helpful in assisting with special case by case referrals, as well as improving communication between referral providers, the GI team, and patients.

Referrals to subspecialty care represent a significant portion of healthcare in this country, and barriers abound. Barriers are arguably more numerous and challenging in large, safety net systems. Given fractured access to healthcare for patients and lack of synergy between systems and providers, our efforts were undertaken to ease these barriers and increase system synergy and efficiency. If successful, this new workflow can be used as an example for other large safety net hospitals to improve their referral system and coordination between the multiple departments that oversee procedures. In Atlanta, with the recent closure of a nearby Level 1 Trauma Center, GHS is seeing increased patient volumes, and thus will benefit greatly from improved referral efficiency. GHS will have a larger influx of new patients and with new facilities opening up GHS’s continued expansion will put our new GI order set to the test. We hope it will serve as a model for similar health care systems in improving triaging of patients and ultimately access to GI patient care. In addition, it can be used as a guide for other departments within GHS as well as other hospital systems at large.

Limitations of this study include the generalizability to other countries and institutions that may not have as many resources such as a robust EMR, IT support, or pre-op anesthesia availability to implement these changes. In addition, staff referring to GI would have an initial learning curve in understanding the new referral workflow.

## Conclusion

We created a new GI order set to improve referrals and ultimately access to GI services at GHS, a large safety net hospital. The Epic order set streamlined the GI referral process and allowed providers to refer patients directly for endoscopic interventions as well as for clinic visits. The new system addressed prior inefficiencies in the workflow, created a novel triage mechanism, and was built to screen for preoperative assessment referral as well as active anticoagulation and antiplatelet medications. An accompanying data dashboard affords access to real time data to identify areas for quality improvement initiatives. We believe this novel order set is a unique innovation and can serve as a model for other GHS departments as well as other hospital systems.

## Supplementary Information

Supplementary Material 1.

## Data Availability

Availability of data and materials: The datasets during and/or analyzed during the current study available from the corresponding author on reasonable request.

## Abbreviations

APP: Advanced practice provider
ASA: American society of anesthesiologists
CRC: Colorectal cancer
EMR: Electronic medical record
FIT: Fecal immunochemical test
GI: Gastroenterology
GHS: Grady Health System
PDSA: Plan-Do-Study-Act
PCP: Primary care provider
PAT: Preoperative anesthesia testing
USPSTF: United States preventive services task force
