# Supplementary material for: Developing user-friendly ambulatory referrals: a quality improvement study in GI referral services at a large academic safety net hospital system
Source: BMC Health Serv Res. 2025 Jul 1;25:832. doi: 10.1186/s12913-025-12976-3 (PMC12211490; doi:10.1186/s12913-025-12976-3)
Supplement: Supplementary file 1 — Supplementary Material 1. [file 12913_2025_12976_MOESM1_ESM.docx]

GI Referral Process Pre-Implementation Survey

1. What is your role within Grady?

- Morehouse Trainee

- Emory Trainee

- Morehouse faculty

- Emory faculty

- Grady Health System employee

- Other (please specify)

1. What is your professional degree?

- Medical Doctor (completed training/board certified)

- Medical Doctor (in fellowship)

- Medical Doctor (in residency)

- Physician Assistant

- Nurse Practitioner

- Other (please specify)

1. In any given month, on average, how many patients do you refer to GI (Gastroenterology services)?

- 0

- 1-2

- 3-5

- >5

1. What is your primary specialty?

- Hospitalist

- Outpatient Primary Care

- OB/Gyn

- Medicine subspecialty

- Neurology/Neuroscience

- Cardiology

- Emergency Medicine

- General Surgery or Surgical Specialist

1. What is the typical nature of your referral? (pick top 1-3)

- Colon Cancer Screening

- Routine General GI

- Urgent General GI

- Routine Subspecialty GI

- Urgent Subspecialty GI

- Routine re-referral for prior established patient that lost continuity

- Urgent re-referral for a prior established patient that lost continuity

1. On average, how many urgent referrals to GI do you make a month?

- 0

- 1-2

- 3-5

- >5

1. In general, how satisfied are you with the timeframe in which your referral will be scheduled, whether urgent or routine?

- Very satisfied

- Somewhat satisfied

- Neither satisfied nor dissatisfied

- Somewhat dissatisfied

- Very dissatisfied

1. In general, to overcome barriers in the referral process, what do you most often do? (pick 1-3)

- Send patient to GI clinic to schedule

- Send patient to GI procedure area to schedule

- Send patient to Emergency Department

- Contact the GI consult pager

- Contact a GI provider

- Ask the patient to call to schedule

- No work around

- Other (please specify)
